# Temporary Shift from Net CO_2_ Uptake to Net CO_2_ Release During the Southwest Monsoon in a Tropical Mangrove Forest

**DOI:** 10.3390/biology15201825

**Published:** 2026-10-13

**Authors:** Maricar Aguilos, Renato Bacsal, Rustum Aguilos, Manuel Anthony P. Jondonero, Michael Angelo Valete, Romell A. Seronay, Eugene Rey Magoncia, Ryan Bacsal, Donald Luna, Nicholas Zeam Ace M. Aguilos, John Benrich Zuniga, Daisy Cabral, Hernando P. Bacosa, Miko Lorenzo Belgado, John S. King

**Affiliations:** 1 Department of Forestry and Environmental Resources, North Carolina State University, Raleigh, NC 27695, USA; mjbelgad@ncsu.edu (M.L.B.); john_king@ncsu.edu (J.S.K.); 2 Forest and Wetland Research, Development and Extension Center, Ecosystems Research and Development Bureau, Bislig 8311, Philippines; renato_bacsal85@yahoo.com (R.B.J.); rustumaguilos1976@gmail.com (R.A.); eugenereym@gmail.com (E.R.M.); ryanbacsal12@gmail.com (R.B.); 3 Natural Sciences Department, Bukidnon State University, Malaybalay City 8700, Philippines; mapjondonero@buksu.edu.ph; 4 Philippine Space Agency (PhilSA), Quezon City 1800, Philippines; michael.valete@philsa.gov.ph; 5 Center for Research in Environmental Management and Eco-Governance (CRÈME), Caraga State University, Ampayon, Butuan City 8600, Philippines; raseronay@carsu.edu.ph; 6 School of Environmental Science and Management, University of the Philippines Los Baños, Los Baños 4031, Philippines; daluna1@up.edu.ph; 7 Caraga Consortium for Agriculture and Resources Research and Development (CCAARRD), Caraga State University, Ampayon, Butuan City 8600, Philippines; nzamaguilos@carsu.edu.ph; 8 Department of Science and Technology- Philippine Council for Agriculture, Aquatic and Natural Resources Research and Development (DOST-PCAARRD), Los Baños 4030, Philippines; j.zuniga@pcaarrd.dost.gov.ph (J.B.Z.); d.cabral@pcaarrd.dost.gov.ph (D.C.); 9 Department of Environmental Science, School of Interdisciplinary Studies, Mindanao State University—Iligan Institute of Technology, Tibanga, Iligan 9200, Philippines; hernando.bacosa@g.msuiit.edu.ph

**Keywords:** eddy covariance, net ecosystem exchange, PhilFlux, Siargao Island, tropical mangrove forest, southwest monsoon, drought

## Abstract

Mangrove forests are important blue-carbon ecosystems, but their atmosphere-surface CO_2_ exchange can vary over short time scales in response to environmental conditions. This study examined CO_2_ exchange in the Del Carmen Mangrove Reserve, Siargao Island, Philippines, using PhilFlux eddy covariance and micrometeorological observations from March 2025 to February 2026. We analyzed diurnal, daily, and monsoon-season patterns of gross primary productivity (GPP), ecosystem respiration (RE), and net ecosystem exchange (NEE). The tower footprint showed net CO_2_ uptake during most of the observation period. August 2025 was the only month with positive aggregated NEE (19 g C m^−2^ month^−1^; 95% CI: −22 g C m^−2^ month^−1^ to 62 g C m^−2^ month^−1^). During August, monthly GPP was 158 g C m^−2^ month^−1^; 95% CI: 130–189 g C m^−2^ month^−1^ and monthly RE was 177 g C m^−2^ month^−1^; 95% CI: 135–226 g C m^−2^ month^−1^. Sixteen of 31 days showed positive NEE, mainly from 8 to 24 August. We therefore interpret this as a temporary positive-NEE episode rather than a month-wide or lasting reversal of CO_2_ uptake. The episode coincided with dry conditions during the Southwest Monsoon. Environmental analyses identified several variables associated with variation in NEE, GPP, and RE, but these relationships are interpreted as statistical associations rather than causal effects. Since the study covers only one annual cycle, longer-term observations are needed to determine whether similar events recur. Continued flux monitoring is needed to better understand the stability of mangrove CO_2_ exchange through time.

## Simple Summary

Mangrove forests remove carbon dioxide (CO_2_) from the atmosphere and store large amounts of carbon, but their CO_2_ exchange can vary with environmental conditions. We examined atmosphere–surface CO_2_ exchange at the Del Carmen mangrove site in Siargao Island, Philippines, from March 2025 to February 2026 using eddy-covariance measurements. During most of the observation period, the tower footprint showed net CO_2_ uptake. In August 2025, however, NEE temporarily shifted toward positive values, indicating reduced CO_2_ uptake and periods of net CO_2_ release within the measured flux footprint. This episode occurred during the Southwest Monsoon and coincided with dry conditions. Since the study covers only one annual cycle, this temporary event should not be interpreted as a lasting loss of the mangrove’s climate benefit or as evidence of a long-term change. Continued flux monitoring is needed to determine whether similar events recur and how they influence longer-term mangrove CO_2_ exchange.

## 1. Introduction

Mangrove forests are among the most carbon-rich ecosystems in the tropics. They protect coastlines, support biodiversity and fisheries, sustain local livelihoods, and help regulate the climate [1,2,3,4]. Their high productivity, waterlogged soils, slow decomposition, and efficient sediment burial allow them to store large amounts of carbon over long periods [1,2,5]. These qualities make mangroves important blue-carbon ecosystems and a growing priority for climate mitigation, coastal adaptation, and restoration.

However, a large carbon stock does not always mean that a mangrove forest consistently functions as a carbon sink. Carbon stock assessments describe long-term storage but cannot capture rapid changes in net ecosystem exchange (NEE), gross primary productivity (GPP), and ecosystem respiration (RE). These fluxes can vary across hours, seasons, and years in response to radiation, temperature, vapor pressure deficit, rainfall, tidal inundation, soil water conditions, and disturbance events [6,7,8,9,10]. At times, respiration may exceed photosynthetic uptake, temporarily shifting the ecosystem from a carbon sink to a carbon source [6,7,8,9,10].

Eddy covariance directly measures ecosystem-scale exchanges of carbon dioxide, water vapor, and energy between mangrove canopies and the atmosphere [11,12]. Flux-tower studies show that mangroves can be strong carbon sinks, but their sink strength changes with atmospheric and hydrological conditions [6,8]. Continuous observations are therefore needed alongside carbon stock assessments to determine not only how much carbon mangroves store, but also how stable their carbon uptake is through time.

Despite their importance, mangroves remain underrepresented in global flux networks. Their saline, tidally inundated, and spatially heterogeneous environments make flux measurements technically challenging. Tower operation and flux interpretation can be affected by unstable substrates, harsh coastal weather, complex canopy structure, and mixed land–water surfaces [6,12]. The tower footprint also changes with wind direction, atmospheric stability, turbulence, measurement height, and surface roughness [13,14,15]. As a result, measured fluxes may represent varying contributions from mangrove vegetation, tidal channels, exposed sediments, and open water. These challenges complicate spatial scaling, but they also make mangrove flux towers valuable for understanding how coastal forests respond to environmental change.

This flux-based information is particularly limited in the Philippines. Most local mangrove carbon studies have focused on biomass, vegetation structure, species composition, and sediment carbon stocks, while direct measurements of NEE, GPP, and RE remain scarce [16,17,18,19]. This leaves an important gap in understanding how mangrove atmosphere-surface CO_2_ exchange varies over short time scales under monsoon, tidal, and drought-related conditions.

The Del Carmen Mangrove Reserve on Siargao Island, Surigao del Norte, provides an important site for addressing this gap. It supports one of the largest contiguous mangrove forests in the Philippines and is recognized for its contributions to coastal protection, biodiversity conservation, livelihoods, and climate resilience [20]. Its climate is influenced by the Northeast Monsoon (*Amihan*), Southwest Monsoon (*Habagat*), the Intertropical Convergence Zone, and tropical cyclone activity. These systems affect rainfall, radiation, humidity, wind, soil water conditions, and atmospheric moisture demand, making Del Carmen a suitable site for examining seasonal changes in mangrove carbon exchange.

The central question of this study is whether atmosphere-surface CO_2_ exchange within a tropical mangrove tower footprint can temporarily shift from net uptake to net release during unusual environmental conditions. However, because this study covers only one annual cycle, it is intended to characterize short-term variability rather than determine whether such events represent a long-term climate trend.

We used PhilFlux eddy covariance and micrometeorological observations from March 2025 to February 2026 to examine the temporal variation in CO_2_ exchange in the Del Carmen mangrove forest. We assessed diurnal, daily, monthly, and monsoon-season patterns of GPP, RE, and NEE, with particular attention to the August 2025 period, when NEE shifted toward positive values. Tower observations were combined with environmental-response modeling and drought-related remote-sensing indicators. Specifically, we asked (1) How do GPP, RE, and NEE vary across diurnal, daily, monthly, and monsoon timescales? (2) Can short-term periods of weakened CO_2_ uptake or temporary net CO_2_ release be detected within the flux tower footprint? (3) Which environmental variables are associated with variations in GPP, RE, and NEE? We hypothesized that (H1) flux tower footprint would show net CO_2_ uptake during most of the study period; (H2) short-term anomalous conditions during the monsoon period would weaken net CO_2_ uptake within the tower footprint; and (H3) daily GPP, RE and NEE would show different associations with radiative, thermal, hydrological, and canopy conditions.

By combining eddy covariance measurements, monsoon-season analysis, remote-sensing indicators, and environmental modeling, this study provides a site-level record of short-term variability in mangrove CO_2_ exchange. The results provide a site-level baseline for longer-term monitoring and help identify when and under what conditions net CO_2_ update may weaken within the tower footprint. Longer records will be needed to determine whether such events are recurring or part of a broader climatic change.

## 2. Materials and Methods

### 2.1. The Study Site

**Figure 1:**
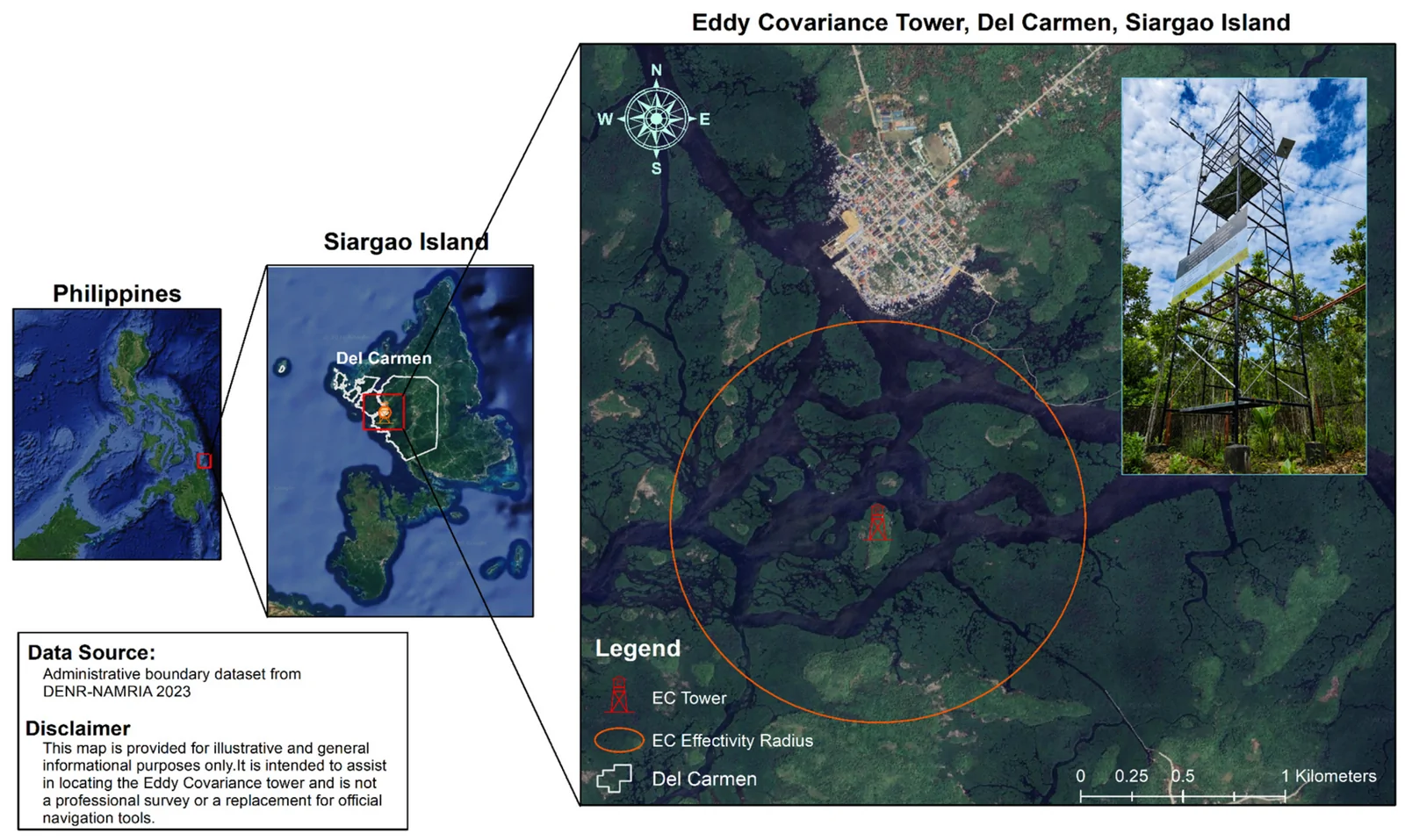
Location of the PhilFlux eddy covariance monitoring tower in the Del Carmen Mangrove Reserve, Siargao Island, Philippines. The tower is indicated by a red marker inside a red circle. The map shows the surrounding mangrove and coastal landscape.

The study was conducted in the approximately 4000-ha Del Carmen Mangrove Reserve within the Siargao Island Protected Landscape and Seascape, Surigao del Norte, Philippines (Figure 1). Located on the western side of Siargao Island, Del Carmen supports one of the country’s largest contiguous mangrove forests. The landscape consists of dense mangrove stands, intertidal flats, shallow marine waters, sandy shorelines, and adjacent coastal habitats. It provides coastal protection from storm surges and strong wave action while supporting resident, migratory, endemic, and threatened wildlife. Its designation as a Ramsar Wetland of International Importance highlights its ecological value and the need for long-term monitoring [20].

The vegetation surrounding the PhilFlux carbon-monitoring tower is dominated by locally common mangrove species, including Bakauan babae (*Rhizophora mucronata*), Bakauan lalaki (*Rhizophora apiculata*), and Busain (*Bruguiera* spp.). These species form structurally complex stands typical of well-developed intertidal mangrove forests. Across the municipality, 20 true mangrove species have been recorded, reflecting the high floral diversity of Del Carmen [21].

Soils in Del Carmen and surrounding areas of Siargao Island are generally clayey, including Bolinao clay and related formations. Low-lying coastal and mangrove areas may also contain Quaternary alluvial deposits of unconsolidated sand, silt, and clay, while sandy loam materials occur closer to shorelines and beach-associated environments [20].

The site has a humid tropical climate. Based on 15-year rainfall records from the General Luna Automated Weather Station, mean annual rainfall is approximately 3757 mm yr^−1^ and mean air temperature of 27.8 °C (Philippine Atmospheric, Geophysical and Astronomical Services Administration) [22]. The station is located approximately 30 km from the PhilFlux tower and was used to represent the broader climatic setting of Del Carmen. In comparison, rainfall measured at the flux tower during the March 2025–February 2026 study period totaled 3570 mm.

Two monsoon systems influence the seasonal climate. The Northeast Monsoon, locally known as *Amihan*, generally occurs from November to March or April and brings cooler northeasterly winds. Since Siargao faces the Pacific Ocean, *Amihan* can also bring rainfall and cloud cover that affect radiation, humidity, wind direction, and surface–atmosphere exchange. The Southwest Monsoon, or *Habagat*, generally occurs from May or June to October and is characterized by warm, moisture-laden southwesterly winds. Tropical cyclones, low-pressure areas, and other weather disturbances can intensify their effects, increasing rainfall, humidity, and cloudiness.

For seasonal comparisons in this study, the Southwest Monsoon (*Habagat*) was defined as 1 May to 30 October 2025. The Northeast Monsoon (*Amihan*) was defined as 16 March to 30 April 2025 and again from 1 November 2025 to 18 February 2026 [22]. The site has a Type II climate, characterized by rainfall throughout the year, no distinct dry season, and more pronounced rainfall from October to February [22].

These seasonal changes can directly influence photosynthesis, ecosystem respiration, evapotranspiration, and net ecosystem exchange. The distinction between *Amihan* and *Habagat* was therefore used to interpret seasonal variations in carbon and water fluxes and identify periods of weakened carbon uptake or temporary carbon release.

### 2.2. Flux and Micrometeorological Measurements

Carbon dioxide, water vapor, and energy fluxes were measured using an eddy covariance system installed on the PhilFlux monitoring tower in the Del Carmen Mangrove Reserve. The main system consisted of a Campbell Scientific IRGASON (Campbell Scientific Inc., Logan, UT, USA), which combines an open-path CO_2_/H_2_O gas analyzer and a three-dimensional sonic anemometer. The sensor was mounted approximately 11 m above ground level on a tower located on a slightly elevated, hilly portion of the mangrove forest. The base of the tower is about 6.5 m above sea level, placing the IRGASON at approximately 17.5 m above sea level. The surrounding mangrove canopy is about 3.5–5 m tall, so the sensor was positioned roughly 6–7.5 m above the canopy. It recorded high-frequency fluctuations in CO_2_, water vapor, three-dimensional wind speed, sonic temperature, air temperature, and barometric pressure. Since the gas analyzer and sonic anemometer are housed in one sensor head, the wind and scalar measurements are co-located and synchronized, helping reduce uncertainties related to sensor separation and time lag [23,24].

Supporting micrometeorological instruments were installed to describe the environmental conditions influencing carbon and water exchange. These included sensors to measure air temperature and compute for relative humidity (%) and vapor pressure deficit (kPa) (HMP 155, LICOR, Inc., Lincoln, NE, USA); rainfall (mm d^−1^, TE-525, Campbell Scientific Inc., Logan, UT, USA), net radiation (W m^−2^ d^−1^, CNR-4, Kipp & Zonen, Delft, The Netherlands), photosynthetic photon flux density (µmol m^−2^ d^−1^, PAR, LI-190, LiCor Inc., Lincoln, NE, USA), soil temperature (°C, CS107, Campbell Scientific, Inc, Logan, UT, USA), soil water content (%, CS616, Campbell Scientific, Inc, Logan, UT, USA), and tidal height (cm d^−1^). These variables were used to interpret changes in NEE, gross primary productivity (GPP), and ecosystem respiration (RE) across daily and monsoon periods. Salinity and independent indicators of biological or physical disturbance were not measured and therefore could not be evaluated directly as possible explanations for the August 2025 event.

### 2.3. Flux Processing, Calculation, and Quality Control

High-frequency data were recorded at 10 Hz and processed using EddyPro v7.0.9 (LICOR Inc, Lincoln, NE, USA). Primary turbulent fluxes were calculated over 30 min averaging intervals, which were used as the primary processing resolution. Standard quality checks and corrections were applied for spike detection [25], where isolated values exceeding approximately 3.5 standard deviations from the local mean and occurring in runs of no more than three consecutive points were treated as spikes. Quality checks were also applied for planar fit coordinate rotation of wind vectors [26], correction of the time lags between scalar concentrations and wind speed (covariance maximization), air density fluctuation [27], and high [28] and low-pass filtering [29].

Flux outputs were assigned quality flags of 0 (high quality), 1 (medium quality) and 2 (low quality) [30]. Fluxes with flags greater than 1 were discarded. Post-processing of the 30 min fluxes included filtering for low signal strength, integral turbulence characteristics, despiking filter, and low friction velocity [31]. Exclusion of the data collected during the non-steady and the low turbulence conditions that were flagged 1 and 2 based on [30] resulted in removal of 2–38% of data from March 2025 to February 2026. August contained 1488 expected half-hourly records, of which 297 were gaps, corresponding to a 19.96% gap fraction (Table 1).

### 2.4. Gap-Filling and Flux Partitioning

Missing and rejected 30 min NEE values were gap-filled using REddyProc v.1.3.2 following the standardized post-processing workflow of Wutzler et al. (2018) [32]. (https://www.bgc-jena.mpg.de/bgi/index.php/Services/REddyProcWeb, accessed on 10 May 2026). The REddyProc online tool implements the standardized methods for processing Eddy-Covariance data. Low-turbulence periods were identified using a site-specific friction-velocity (u*) threshold estimated from nighttime NEE–u* relationships across temperature and seasonal classes. One hundred bootstrap samples were used to characterize uncertainty in ustar (u*) and the 5th, 50th, and 95th percentile thresholds were retained as alternative processing scenarios. The median (u*) threshold was used for the main analysis, while the lower and upper thresholds were used to assess sensitivity of the resulting flux estimates. REddyProc then gap-filled NEE using Marginal Distribution Sampling, which estimates missing fluxes from observations under similar meteorological conditions and from mean diurnal patterns when necessary [33].

The gap-filled 30 min NEE series was partitioned into gross primary productivity (GPP) and ecosystem respiration (RE) using the nighttime-based approach implemented in ReddyProc [33]. Under the nighttime method, the temperature dependence of nighttime respiration is estimated and extrapolated to daytime periods, and GPP is calculated from the difference between modeled RE and NEE. Thus, GPP and RE are model-derived components of NEE and were not treated as independent flux measurements.

Gap length and data coverage were recorded before gap filling. REddyProc was used for the main gap-filling procedure. Gap filling and flux partitioning using REddyProc left only a small number of remaining gaps (<2%) in the NEE, GPP, and RE time series. These short residual gaps were filled by linear interpolation using the valid 30 min values immediately before and after each missing observation.

The 30 min NEE record was used as the base time series for all temporal aggregations. Hourly NEE was calculated by summing two consecutive 30 min NEE values. Daily NEE was calculated by summing all 48 half-hourly values within each 24 h period, and monthly aggregated NEE was calculated by summing the corresponding daily fluxes over each month. Carbon balance was reported using the atmospheric sign convention, where positive NEE indicates a net loss of carbon from the ecosystem to the atmosphere, while negative NEE indicates net ecosystem carbon uptake [34].

### 2.5. Flux Tower Footprint Analysis

The tower measurements represent the integrated fluxes within the eddy covariance footprint, which varies with wind direction and atmospheric stability. Thus, the results were interpreted as ecosystem-scale fluxes for the tower footprint rather than direct estimates for the entire Del Carmen Mangrove Reserve. Flux footprint analysis was conducted following the process flow described by Kljun et al. (2015) [35]. Footprint estimates were used to identify the likely source areas contributing to the measured fluxes. Annual and monthly footprint distributions were mapped relative to the tower using cumulative contribution contours from 10% to 90% at 10% intervals. The footprint varied in size and orientation through time, reflecting changes in wind direction and atmospheric conditions. The maps were used to assess whether the measured fluxes were dominated by the surrounding mangrove canopy or whether nearby tidal channels, open water, and exposed sediment may also have contributed to the observed exchange.

### 2.6. Data Processing

Flux and micrometeorological data were summarized at hourly, daily, monthly, and seasonal time scales to describe temporal patterns in carbon and water exchange. Seasonal comparisons were made between the Northeast Monsoon (*Amihan*) and Southwest Monsoon (*Habagat*) periods to examine whether monsoon conditions influenced changes in NEE, GPP, and RE. Daily NEE, GPP, and RE were used as response variables, and environmental predictors were aggregated to the same daily time step. Only observations with complete data for the response and candidate predictors were included in model selection, resulting in 257 complete daily observations. An initial set of 13 environmental variables was considered: albedo, high tide, low tide, rainfall, PPFD, relative humidity, net radiation, soil water content, air temperature, soil temperature, VPD, wind speed, and LAI. These variables were first screened for multicollinearity using variance inflation factors (VIF), with a cutoff of 5. Variables were removed sequentially beginning with the highest VIF. The *caTools* and *car* packages in R were used [36,37]. PPFD and VPD were removed during this step, leaving 11 candidate predictors with VIF values below 5.

The remaining 11 predictors were then evaluated separately for NEE, GPP, and RE using bidirectional stepwise selection based on Akaike’s Information Criterion (AIC) [38]. Predictors retained by AIC were included in generalized additive models (GAMs) using the *mgcv* package in R to examine possible nonlinear relationships with the daily fluxes. The same procedure was applied to the full-period dataset and the August-only dataset.

Model adequacy was assessed using residual plots and residual autocorrelation functions. Model performance was summarized using adjusted *R*^2^ and temporally blocked cross-validation, including root mean square error (RMSE). F-statistics and *p*-values for GAM smooth terms were used only to test whether individual smooth terms contributed to the model and were not interpreted as measures of causal importance or directly comparable predictor effect sizes.

Since GPP and RE were obtained by partitioning NEE using environmental-response models, analyses of GPP and RE were interpreted cautiously. Relationships between environmental variables and partitioned GPP or RE may partly reflect the assumptions used during flux partitioning and should therefore be considered statistical associations rather than independent causal responses.

Environmental conditions during August were also compared with adjacent months to determine whether the positive-NEE episode coincided with changes in atmospheric, hydrological, or canopy conditions. These comparisons were treated as descriptive and associative rather than as tests of causation.

All analyses and visualizations were conducted in R using, e.g., *tidyverse*, *ggplot2*, *reshape*, and other packages [39,40,41].

### 2.7. Drought Detection

Meteorological drought conditions were characterized using the Standardized Precipitation Index at a 3-month timescale (SPI-3) [42]. SPI-3 was selected to represent short-term precipitation variability and seasonal moisture conditions relevant to vegetation response. Daily rainfall data from the Global Precipitation Measurement (GPM) Integrated Multi-satellite Retrievals for GPM (IMERG) Version 07 Daily Late Run product [42], at 0.1° × 0.1° spatial resolution (aggregated to monthly totals for SPI-3 calculation) were obtained from the NASA Goddard Earth Sciences Data and Information Services Center (GES DISC) for January 2001 to February 2026. Monthly rainfall totals were calculated only for months with complete daily coverage; incomplete months were excluded from SPI fitting and ranking.

SPI-3 was calculated following the procedure described in [42] using 3-month accumulated monthly precipitation. For each calendar month, the gamma-distribution parameters were estimated separately using all available observations for that calendar month within the January 2001–February 2026 record. The probability of zero precipitation was incorporated into the cumulative probability distribution, which was subsequently transformed to the standard normal distribution to obtain SPI values. Thus, the SPI distribution was calendar-month specific; for example, August SPI-3 values were evaluated against a distribution fitted using the available August 3-month precipitation accumulations from 2001–2025.

A point-to-pixel approach was used to characterize precipitation conditions at the PhilFlux tower location. The IMERG grid cell containing the tower was selected, with a grid-cell center at approximately 125.9500° E, 9.8500° N. This pixel-level extraction was used to derive the SPI-3 time series associated with the tower site.

Vegetation condition was assessed using the Normalized Difference Vegetation Index (NDVI) from the Moderate Resolution Imaging Spectroradiometer (MODIS) Terra Vegetation Indices Monthly product (MOD13C2 Version 6.1) at 0.05° × 0.05° spatial resolution. Monthly NDVI data covering January 2001 to February 2026 were used to characterize temporal variations in vegetation condition. Only MOD13C2 observations meeting the specified quality criteria, corresponding to QA values 00 and 01, were retained.

To account for seasonal variability and facilitate interannual comparison, NDVI was standardized separately for each calendar month. The standardized NDVI anomaly was calculated as:

$${Standarzied\;}_{NDVI}=\;\frac{NDVI-\bar{\;{NDVI}_{m}}}{\sigma_{m}}$$

where the *NDVI* is the observed monthly NDVI, *NDVI_m_* is the climatological mean for the corresponding calendar month, and the σ_m_ is the corresponding standard deviation. The climatology was calculated from the available January 2001–February 2026 record. The MOD13C2 pixel corresponding to the tower site was selected using a point-to-pixel approach and has a grid-cell center at approximately 125.9750° E, 9.8750° N. Negative standardized NDVI values indicate below-normal vegetation greenness relative to the historical record, whereas positive values indicate above-normal vegetation conditions.

The selected IMERG and MOD13C2 pixels include the PhilFlux tower location, al-lowing the satellite-derived indicators to be related to the tower observations. However, the satellite pixels have fixed spatial extents, whereas the eddy covariance footprint varies with environmental and atmospheric conditions. Therefore, SPI-3 and standardized NDVI were interpreted as indicators of conditions at the tower site rather than direct measurements of the vegetation within the instantaneous flux footprint.

## 3. Results

### 3.1. Daily Climate Patterns

**Figure 2:**
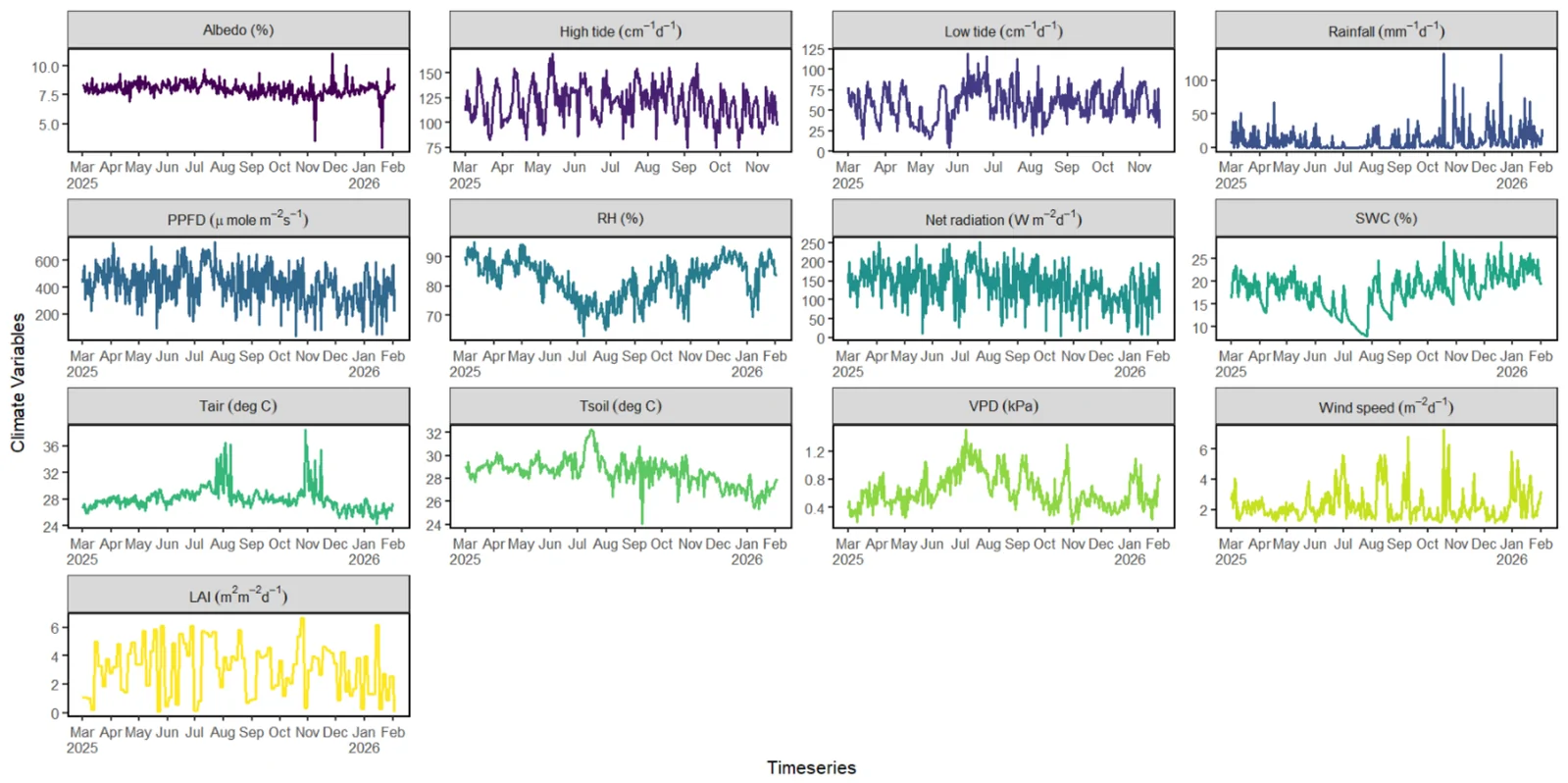
Climatic trends from March 2025 to February 2026 at the PhilFlux tower in Del Carmen, Siargao Island, Philippines. Daily data were used in the analysis.

Meteorological and site conditions at the Del Carmen PhilFlux tower varied substantially from March 2025 to February 2026 (Figure 2). Average daily albedo was 7.99%, with values ranging from 2.95% to 11.09%. Tidal conditions also showed wide daily variation. High tide ranged from 75 to 190 cm, with a mean of 119 cm. Low tide ranged from 5 to 120 cm, with a mean of 58 cm. Rainfall totaled 3570 mm during the study period, with several rain-free days and daily rainfall reaching up to 140 mm. Photosynthetic photon flux density (PPFD) varied from 36.95 umol m^−2^ s^−1^ to 742.55 umol m^−2^ s^−1^ (mean 420.86 umol m^−2^ s^−1^). Relative humidity (RH) ranged from 62.95% to 95.27%, with a mean of 82.51%. Mean net radiation (Rn) was 140 W m^−2^ (varied from 4.99 W m^−2^ to 253.05 W m^−2^. Soil water content (SWC) ranged from 7.99% to 28.80%, with a mean of 18.31%. Air temperature ranged from 24.35 to 38.51 °C, with a mean of 28.13 °C. Soil temperature showed a narrower range, from 24.06 to 32.56 °C, with a mean of 28.53 °C. Average vapor pressure deficit (VPD) was 0.64 kPa, ranging from 0.17 kPa to 1.50 kPa. Wind speed varied from 1.07 to 7.27 m s^−1^, with an average of 2.28 m s^−1^. Leaf area index ranged from 0.04 to 6.66 m^2^ m^−2^, with a mean of 3.09 m^2^ m^−2^.

### 3.2. Diurnal Cycle of Carbon Exchange

**Figure 3:**
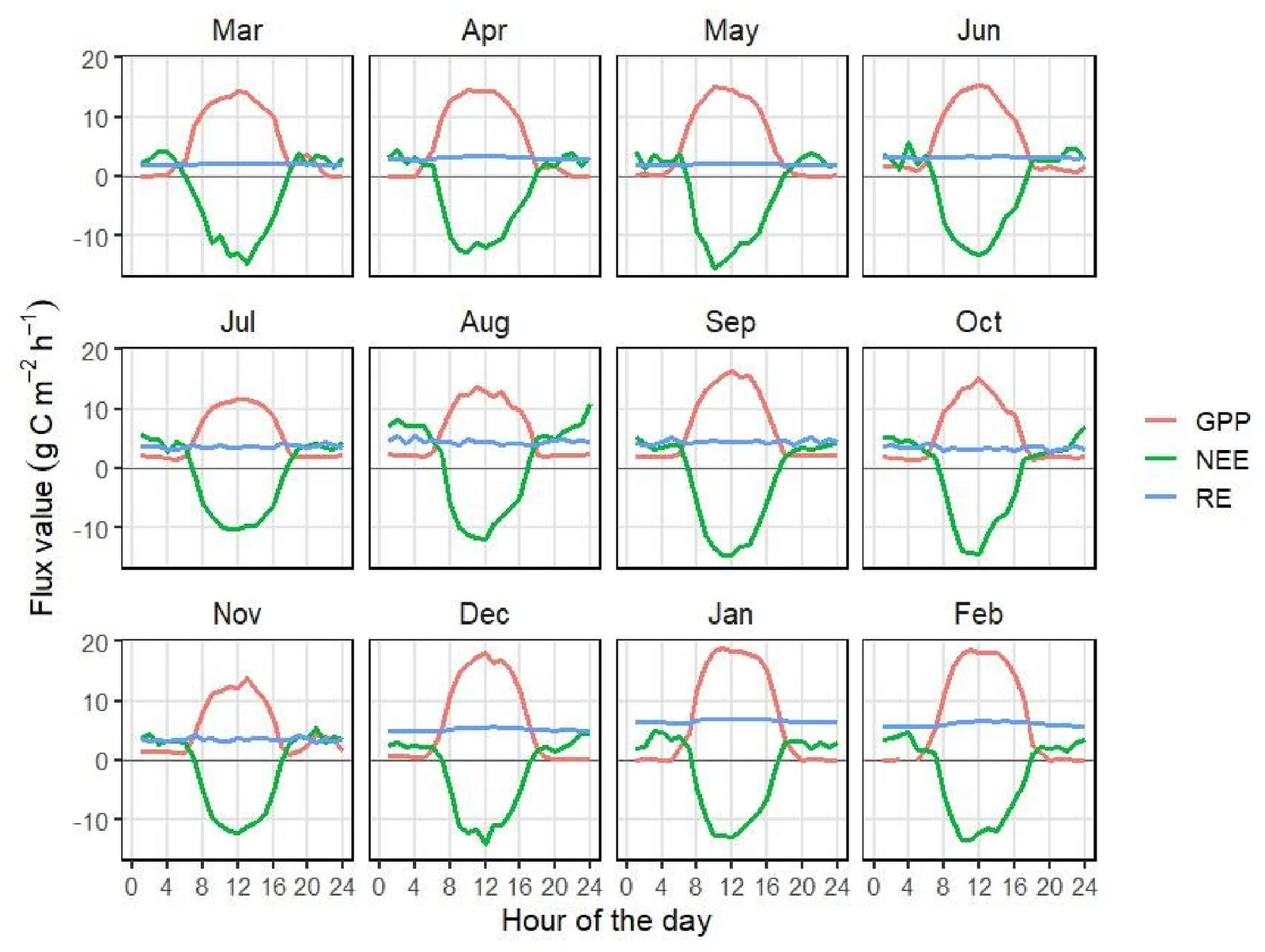
Diurnal pattern of gross primary productivity (GPP), ecosystem respiration (RE), and net ecosystem exchange (NEE) from March 2025 to February 2026 at the PhilFlux tower in Del Carmen, Siargao Island, Philippines. Hourly data were used in the analysis.

The Del Carmen Mangrove Reserve showed a clear diurnal pattern of carbon exchange from March 2025 to February 2026 (Figure 3). Gross primary productivity (GPP) increased after sunrise, reached its highest values near midday, and declined toward zero at night. Mean GPP was 6.30 g C m^−2^ h^−1^. Ecosystem respiration (RE) remained positive throughout the day and was comparatively stable, with a mean of 3.99 g C m^−2^ h^−1^.

Net ecosystem exchange (NEE) followed the daily balance between GPP and RE. Mean NEE was −1.91 g C m^−2^ h^−1^. During daylight, NEE generally became negative, indicating net CO_2_ uptake by the mangrove canopy. At night, NEE shifted to positive values, indicating net CO_2_ release from ecosystem respiration. This diel pattern was observed across most months, showing that the forest maintained active carbon uptake during daylight periods. The magnitude of the diurnal cycle varied among months. Months with higher GPP showed stronger daytime carbon uptake and deeper negative NEE values. In contrast, transitional or disturbance periods showed weaker daytime uptake, with NEE approaching neutral or becoming positive. These results show that seasonal changes mainly affected the strength of carbon uptake and release, while the general timing of the diurnal cycle remained consistent.

### 3.3. Daily and Seasonal Carbon Balance and Temporary Source Transition

**Figure 4:**
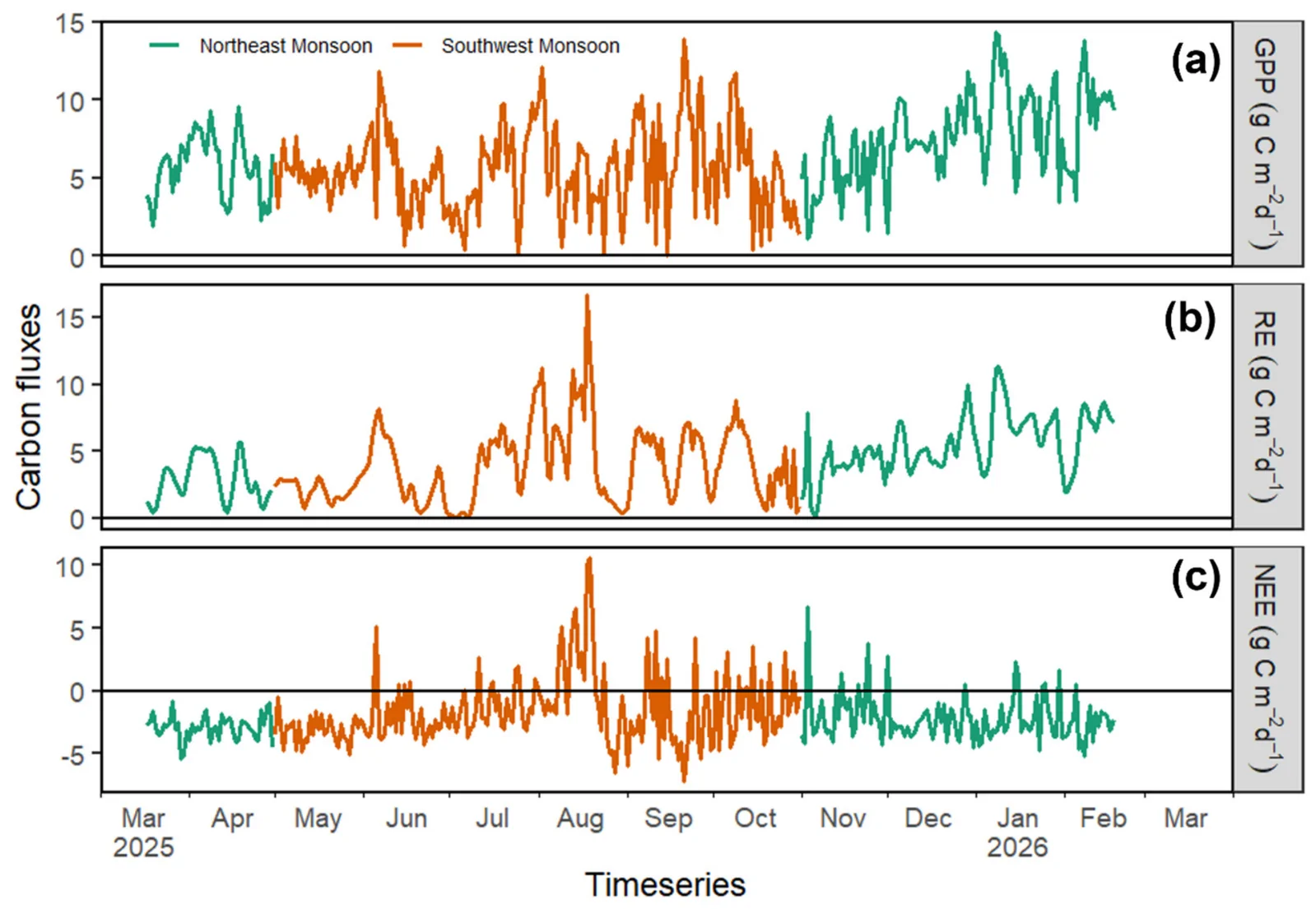
Daily changes in (**a**) gross primary productivity (GPP), (**b**) ecosystem respiration (RE), and (**c**) net ecosystem exchange (NEE) from March 2025 to February 2026 at the PhilFlux tower in Del Carmen, Siargao Island, Philippines. For seasonal comparison, the Southwest Monsoon (*Habagat*) was defined as 1 May to 30 October 2025, while the Northeast Monsoon (*Amihan*) was defined as 16 March to 30 April 2025 and 1 November 2025 to 18 February 2026. Daily data were used in the analysis.

Daily carbon fluxes showed that the Del Carmen mangrove forest acted as a net carbon sink for most of the study period (Figure 4c). Mean daily GPP was 6.23 g C m^−2^ d^−1^, 95% CI: 5.92 g C m^−2^ d^−1^–6.54 g C m^−2^ d^−1^ (Table 2), while mean daily NEE was approximately −1.94 g C m^−2^ d^−1^, 95% CI: −2.19 g C m^−2^ d^−1^ to −1.67 g C m^−2^ d^−1^ under the atmospheric sign convention. This indicates that photosynthetic carbon uptake generally exceeded ecosystem respiratory losses (mean 4.29 g C m^−2^ d^−1^, 95% CI: 4.01 g C m^−2^ d^−1^–4.57 g C m^−2^ d^−1^) (Table 2).

A pronounced weakening of the carbon sink occurred during the mid-year period, with the clearest departure observed in August 2025. August was the only month in the study period with a positive aggregated NEE (Table 2). Mean daily NEE was 0.60 g C m^−2^ d^−1^, with a 95% confidence interval of −0.72 g C m^−2^ d^−1^ to 2.02 g C m^−2^ d^−1^, while the monthly NEE sum was 19 g C m^−2^ month^−1^, 95% CI: −22 g C m^−2^ month^−1^ to 62 g C m^−2^ month^−1^. Daily analysis showed that 16 of the 31 August days had positive NEE, compared with 15 days with negative NEE (Table 2), and the positive exchange was concentrated between 8 and 24 August. Thus, the observed source behavior was not sustained across every day of the month but occurred as a sustained temporary positive-NEE episode within August. Figure 4c shows this mid-August shift toward positive NEE, followed by a return toward negative values afterward.

By September 2025, NEE had returned to predominantly negative values, indicating recovery of net ecosystem carbon uptake. Overall, the daily time series indicates that the mangrove forest remained a carbon sink for most of the study period but experienced a distinct temporary positive-NEE episode during August.

### 3.4. Flux Footprint Analysis Results

**Figure 5:**
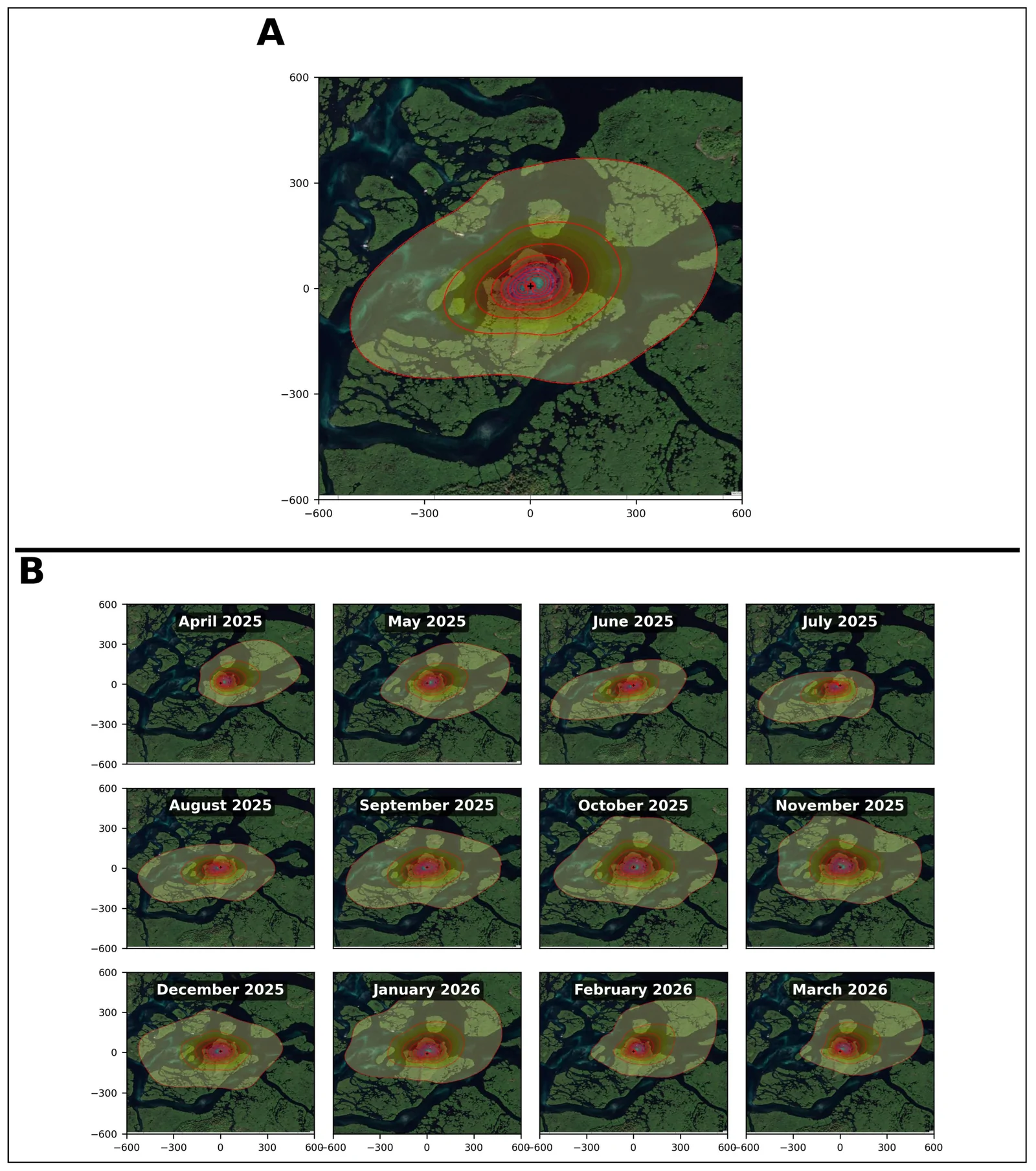
Annual (**A**) Annual and monthly flux footprints from April 2025 to March 2026. (**A**) Annual flux footprint and (**B**) monthly flux footprints showing the spatial distribution of source areas contributing to the measured fluxes. The semi-transparent light-green shading represents the flux footprint, while the red contour lines indicate cumulative flux contributions from 10% to 90% at 10% intervals. The underlying satellite imagery shows surrounding vegetation (green) and waterways (dark blue to black). The plus sign (+) marks the eddy covariance tower location, and the x- and y-axes indicate distances from the tower in meters (m).

The annual and monthly footprint maps showed that the main source area was generally concentrated around the mangrove stand surrounding the tower, although the footprint changed in extent and direction among months (Figure 5). The outer footprint contours occasionally extended over nearby tidal channels, open water, and possible exposed sediment. The August 2025 footprint remained broadly centered over the mangrove area and did not show an obvious shift to a substantially different source area compared with adjacent months.

### 3.5. Monsoon-Season Response

During the Northeast Monsoon (*Amihan*) period, NEE remained predominantly negative, indicating that the mangrove forest functioned as a net carbon sink (Figure 4c). In contrast, during the Southwest Monsoon (*Habagat*) period, NEE increased toward zero beginning in June 2025, with the largest departure observed in August 2025 (Figure 4c).

August 2025 showed a noticeable departure from the prevailing carbon-exchange pattern during the measurement period. It was the only month with a positive mean daily NEE and positive monthly aggregated NEE, with a mean daily value of 0.60 g C m^−2^ d^−1^, 95% CI: −0.72 g C m^−2^ d^−1^ to 2.02 g C m^−2^ d^−1^ and a monthly sum of 19 g C m^−2^ month^−1^, 95% CI: −22 g C m^−2^ month^−1^ to 62 g C m^−2^ month^−1^ (Table 2). However, the 95% confidence interval of −0.72 g C m^−2^ d^−1^ to 2.02 g C m^−2^ d^−1^, for the mean daily NEE indicates uncertainty. At the daily scale, 16 days had positive NEE and 15 days had negative NEE, with the positive exchange concentrated between 8 and 24 August. This pattern is also evident in Figure 4c, where NEE shifted toward positive values during early to mid-August before returning toward negative values. We therefore interpret the event as a sustained temporary positive-NEE episode within August, rather than a continuous month-wide transition to a carbon source. August contained all 31 valid days, with 1488 expected half-hourly observations and 297 data gaps, corresponding to 19.96% gap-related uncertainty (Table 1).

**Figure 6:**
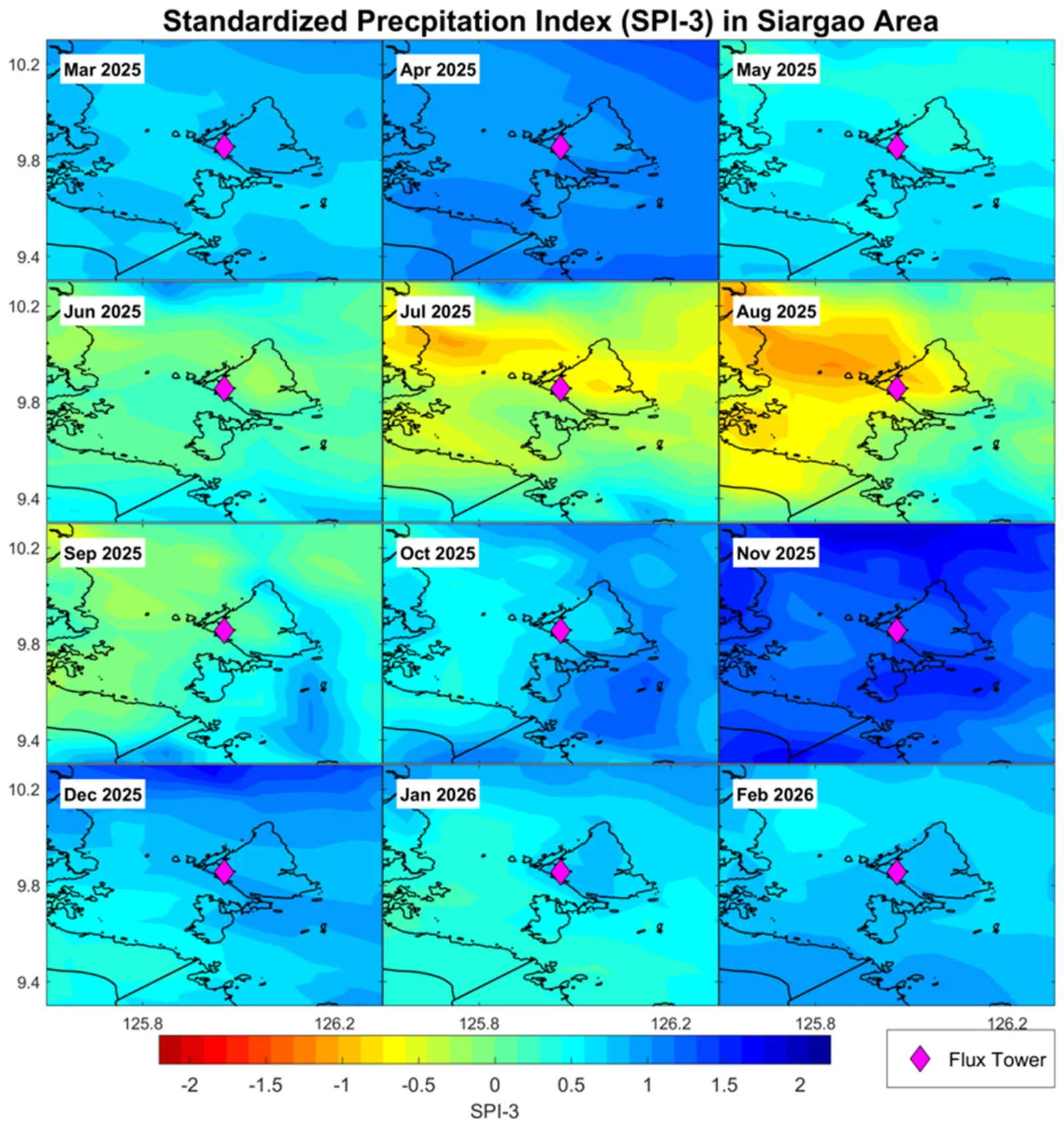
Monthly SPI-3 maps of the Siargao study area from March 2025 to February 2026. Relatively drier conditions were observed during August 2025, while wetter conditions prevailed during November 2025 to February 2026.

**Figure 7:**
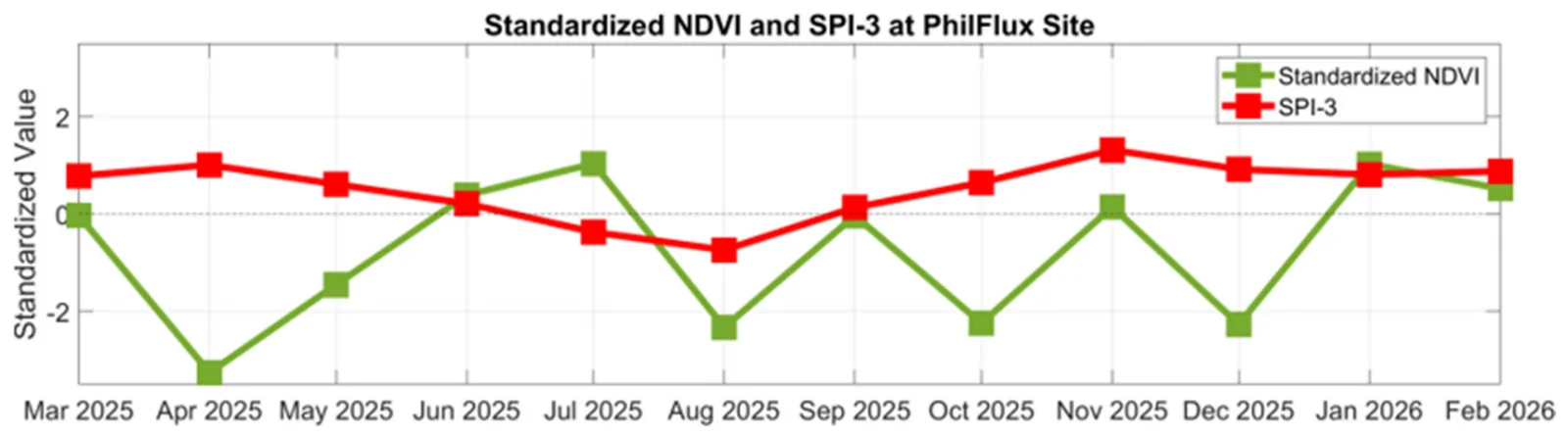
Temporal variation in SPI-3 and standardized NDVI at the PhilFlux mangrove site from March 2025 to February 2026. Periods of negative standardized NDVI indicate below-normal vegetation condition, while SPI-3 reflects short-term meteorological moisture conditions.

**Figure 8:**
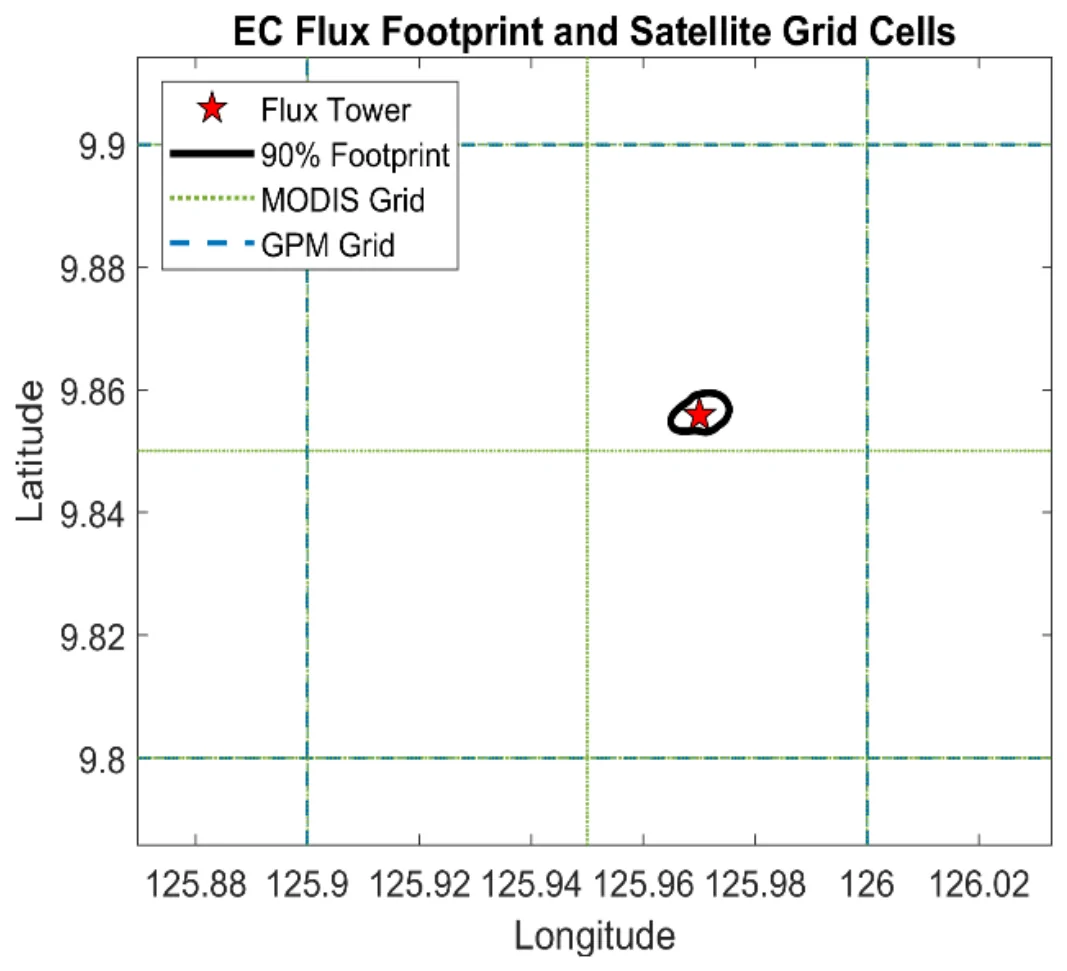
Spatial relationship among the PhilFlux tower, the selected GPM IMERG precipitation grid cell, the selected MOD13C2 NDVI grid cell, and the August 2025 eddy-covariance footprint. The satellite pixels provide environmental context for the tower site but do not correspond exactly to the dynamic flux footprint.

At the PhilFlux site, the August 2025 SPI-3 was −0.74, indicating mild drought conditions (Figure 6 and Figure 7). This was the seventh-lowest August SPI-3 value in the 2001–2025 record, with six August values being lower. Thus, August 2025 was relatively dry during the study period but was not historically exceptional (Figure 6). Standardized NDVI at the corresponding MODIS pixel was −2.33, indicating substantially below-normal vegetation greenness relative to historical August conditions (Figure 7). These satellite-derived anomalies coincided with the temporary positive-NEE episode. Since SPI-3 and NDVI were derived from fixed satellite pixels rather than the instantaneous tower footprint (Figure 5 and Figure 8), these values are interpreted as site-level environmental indicators rather than direct measurements of conditions within the flux footprint.

### 3.6. Environmental Associations with Daily Carbon Fluxes

Multicollinearity screening removed PPFD and VPD from the original candidate predictor set using a VIF threshold of 5. The remaining variables were evaluated using bidirectional AIC selection and generalized additive models. A common complete-case dataset of 257 daily observations for the entire duration and 31 observations for August were only used during model selection (Table 3).

For the dataset covering the whole duration, the selected predictor set for NEE included rainfall, relative humidity, albedo, soil temperature, soil water content, and high tide (Table 3). The corresponding final GAM had an adjusted R^2^ of 0.38. For GPP, the retained variables were soil temperature, albedo, rainfall, wind speed, soil water content, and LAI, with an adjusted R^2^ of 0.20. For RE, the retained variables were relative humidity, net radiation, soil temperature, LAI, rainfall, and albedo, with an adjusted R^2^ of 0.19. Temporally blocked cross-validation indicated limited predictive performance. RMSE values were 2.53 for NEE, 2.61 for GPP, and 2.44 for RE (Table 3). These results indicate that the models were more useful for describing environmental associations within the observed dataset than for predicting fluxes outside the fitted periods.

For the August-only analysis, the same 11 candidate predictors remaining after VIF screening were evaluated using bidirectional AIC selection (Table 3). For NEE, air temperature, wind speed, and low-tide height were retained, with an adjusted R^2^ of 0.61. GPP was associated with soil temperature, air temperature, relative humidity, soil water content, and low-tide height (adjusted R^2^ = 0.72), while RE retained wind speed, low-tide height, relative humidity, soil temperature, and LAI (adjusted R^2^ = 0.60). Temporally blocked cross-validation produced RMSE values of 2.26, 2.20, and 2.75 for NEE, GPP, and RE, respectively. These August-only models indicate that the positive-NEE episode coincided with multiple atmospheric, hydrological, and canopy-related conditions rather than being attributable to a single environmental variable. While this August-specific analysis identified several environmental variables associated with the positive-NEE episode, however, the models did not test statistical interactions and do not establish that these factors jointly caused the event.

## 4. Discussion

### 4.1. Diurnal and Daily Patterns of GPP, RE, and NEE

Carbon exchange in the Del Carmen mangrove forest followed a clear diurnal pattern. During daylight, photosynthetic uptake increased and NEE became negative, indicating net CO_2_ uptake. At night, photosynthesis stopped while ecosystem respiration continued, causing NEE to become positive and indicating net CO_2_ release. This pattern is typical of eddy-covariance measurements in mangrove and other forest ecosystems, where light strongly controls daytime carbon uptake while respiration occurs throughout the day and night [8,10,11,12,43].

The observed pattern is consistent with findings from the Sundarbans mangrove forest in India and subtropical mangroves in southern China, where daytime uptake and nighttime respiratory release determined the daily carbon balance [10,12]. However, these studies also showed that the strength of carbon uptake varied with seasonal and interannual environmental conditions. Del Carmen exhibited the same general behavior, with a consistent diurnal cycle but changing uptake strength through time.

At the daily scale, GPP generally exceeded RE, allowing the forest to function as a net carbon sink during most of the measurement period. However, this sink strength was not constant. The difference between GPP and RE narrowed during the middle of the year and culminated in the August 2025 anomaly, when net carbon uptake weakened substantially. This response shows that the carbon balance of the forest was sensitive to short-term atmospheric and hydrological variability. Overall, these results support H1. Net CO_2_ uptake was observed within the tower footprint during most of the study period, although its strength varied through time. This indicates that the mangrove footprint generally acted as a CO_2_ sink during the measurement period, while also showing substantial short-term variability.

### 4.2. August 2025 Temporary Positive-NEE Episode During the Southwest Monsoon

The most notable feature of the annual record was the temporary shift in NEE toward positive values in August 2025. Under the atmospheric sign convention used in this study, positive NEE indicates that the ecosystem released more CO_2_ than it absorbed. The event occurred during the Southwest Monsoon (*Habagat*) and coincided with unusual environmental conditions.

Two lines of evidence support this interpretation. First, the tower record showed several days with positive or near-positive NEE, indicating that GPP had weakened relative to RE (Figure 4). Second, SPI-3 indicated mild drought conditions, providing independent evidence of short-term moisture stress (Figure 6 and Figure 7). Together, these findings suggest that the anomaly was not simply a random fluctuation within the tower record.

The August-only analysis provides additional context for the temporary positive-NEE episode. Using the same 11 environmental variables retained after VIF screening for the full study period, the August models identified associations with several atmospheric, hydrological, and canopy-related variables. Daily NEE was associated with air temperature, wind speed, LAI, and low-tide height, while GPP was associated with air and soil temperature, relative humidity, soil water content, and low-tide height. RE was associated with wind speed, low-tide height, relative humidity, soil temperature, and LAI. The August models explained 61%, 72%, and 60% of the adjusted variance in NEE, GPP, and RE, respectively, although the small August sample size (n = 31) requires cautious interpretation.

Importantly, these results do not identify a single cause of the August positive-NEE episode. Instead, they indicate that the event occurred under a combination of atmospheric, hydrological, tidal, and canopy conditions. The retention of low-tide height and wind speed in the August models also supports consideration of mechanisms other than drought alone, including changes in inundation and source-area contributions. Thus, the mild SPI-3 drought and negative NDVI anomaly should be interpreted as part of the environmental context of the event rather than as direct evidence that drought caused the temporary shift toward positive NEE.

Mangrove carbon exchange is sensitive to radiation, temperature, inundation, and water availability [6,8,9,10]. Drought and high atmospheric moisture demand can constrain canopy photosynthesis, while ecosystem respiration may remain active. This imbalance weakens net carbon uptake and can temporarily shift the ecosystem toward net CO_2_ release [44,45,46]. The August event coincided with lower GPP relative to RE and mild drought conditions during the *Habagat* season, but these observations do not establish the mechanism responsible for the event.

Although the available evidence cannot attribute the event to a single driver, the tower measurements and SPI-3 results provide environmental context for the August anomaly, but they do not establish a climate-related cause. The result shows that net atmospheric CO_2_ uptake within the tower footprint can weaken or temporarily shift toward net CO_2_ release under unfavorable environmental conditions. These results show that mangrove CO_2_ exchange can vary over short periods under changing environmental conditions.

The August episode coincided with relatively dry conditions and changes in several measured environmental variables, but these observations do not establish a single causal mechanism. Footprint variability and data-processing choices must also be considered. The August footprint remained broadly centered over the mangrove stand and did not show an obvious shift to a substantially different source area, although varying contributions from tidal channels, open water, and exposed sediment cannot be excluded. Likewise, gap filling, u* filtering, and partitioning of NEE into GPP and RE introduce uncertainty into the magnitude of the derived flux components. We therefore interpret the climatic conditions as environmental context for the event rather than as proof of causation.

Overall, the August-specific analysis supports a multivariable interpretation of the positive-NEE episode and does not support attribution to drought or monsoon conditions alone.

The August 2025 episode provides partial support for H2. Net CO_2_ uptake weakened and temporarily shifted toward positive NEE during the Habagat period, coinciding with unusual environmental conditions. However, because the study covers only one annual cycle, these observations show short-term variability rather than a general or causal monsoon effect. The footprint analysis also showed that the August source area remained broadly comparable with adjacent months, reducing the likelihood that the anomaly resulted only from a major change in footprint location.

### 4.3. Interpreting the August 2025 Anomaly in a Single-Year Record

The August 2025 temporary positive-NEE episode shows that atmospheric CO_2_ uptake within the tower footprint can be temporarily reduced or reversed over short periods. However, because the dataset covers only one annual cycle, the event cannot yet be interpreted as a long-term trend or direct signal of climate change. It is better viewed as a short-term anomaly observed under variable environmental conditions.

Long-term eddy-covariance studies have shown that mangrove carbon and water fluxes vary substantially among seasons and years in response to environmental conditions [8,9,10,47]. A single anomalous event cannot establish a new pattern, but it can reveal how the ecosystem responds to stress. Longer-term observations are needed to determine whether similar events recur under drought, heat, rainfall variability, or unusual monsoon conditions, and whether they affect the annual carbon balance.

Continued monitoring is therefore needed to determine whether the August event was rare or part of a recurring response. A multi-year record would allow comparisons among typical monsoon years and years affected by drought, tropical cyclones, or other climate anomalies. It would also help distinguish short-term meteorological effects from longer-term changes in canopy condition, species composition, hydrology, and disturbance exposure. The key question is not only whether Del Carmen functions as a carbon sink, but how stable that sink remains under a changing climate.

### 4.4. Environmental Associations with Daily NEE, GPP, and RE

Daily carbon fluxes were associated with several radiative, thermal, hydrological, and canopy variables. Their relative associations differed among GPP, RE, and NEE, showing that each flux component responded to a distinct but connected set of environmental conditions [6,7,8,9].

The statistical analyses identified several environmental variables that were associated with daily variation in NEE, GPP, and RE, including radiation, temperature, precipitation, soil moisture, wind speed, LAI, and tidal conditions as observed in other mangrove forests [48,49,50]. These relationships are consistent with the known sensitivity of mangrove carbon exchange to atmospheric, hydrological, and canopy conditions [6,8]. Ecosystem respiration is known to be temperature sensitive, although hydrology-related variables can modify respiration by affecting soil saturation, oxygen availability, root activity, and decomposition [51,52,53].

However, the models should not be interpreted as showing that any individual variable caused the observed flux changes. The limited performance under temporally blocked cross-validation further indicates that the models primarily describe associations within the observed study period rather than provide strong predictive relationships.

Interpretation of the GPP and RE models requires additional caution because these fluxes were not independently measured. Both were derived from NEE through the REddyProc partitioning procedure, which itself uses environmental-response relationships. Consequently, some of the statistical associations between environmental variables and GPP or RE may partly reflect the assumptions used during partitioning. For this reason, we focus on the overall shapes and uncertainty of the fitted responses rather than ranking predictors according to their F-statistics.

The August 2025 positive-NEE episode was therefore evaluated using the observed daily flux pattern together with environmental conditions rather than relying on the GAMs as evidence of causation. The statistical models provide supporting context for the environmental conditions associated with flux variability, but they do not independently establish the mechanism responsible for the August anomaly.

The results provide partial support for H3. Daily NEE, GPP, and RE were associated with several radiative, thermal, hydrological, and canopy variables, broadly consistent with our expectations. However, the specific relationships differed among flux components, and the models should be interpreted as showing statistical associations rather than causal effects. In addition, GPP and RE were derived through flux partitioning and were not independent measurements.

Taken together, H1 was supported by the predominance of net CO_2_ uptake during the study period, while H2 and H3 received partial support. The August episode showed that CO_2_ uptake can weaken over short periods, and the environmental analyses identified several associated conditions, but the one-year record does not allow broader causal or climatic conclusions.

### 4.5. Implications

This study provides direct observations of atmosphere–surface CO_2_ exchange in a tropical mangrove setting that remains poorly represented in global eddy-covariance networks. The Del Carmen PhilFlux tower therefore adds useful information on how mangrove CO_2_ exchange varies across daily and seasonal conditions.

The results also show the value of combining tower measurements with micrometeorological observations, drought indicators, vegetation information, and footprint analysis. Together, these datasets help place short-term changes in NEE within their environmental and spatial context. However, because the study covers only one annual cycle, the observed August 2025 positive-NEE episode should be viewed as a short-term anomaly rather than evidence of a persistent change in mangrove carbon function.

These findings are relevant for long-term mangrove monitoring and blue-carbon research because they show that atmospheric CO_2_ uptake can vary substantially over short periods. At the same time, eddy covariance measures CO_2_ exchange within a changing tower footprint and does not represent the complete carbon balance of the entire reserve. Longer records, together with measurements of other carbon pathways, are needed before these observations can be used to assess long-term carbon sequestration or broader climate responses.

### 4.6. Uncertainty and Limitations

This study covers only one annual cycle, limiting our ability to determine whether the August 2025 source event was rare or recurring. It should therefore be interpreted as a short-term disturbance signal rather than evidence of a long-term trend.

Eddy-covariance measurements represent a changing tower footprint rather than the entire mangrove reserve. Although the August footprint remained broadly centered over mangrove vegetation, changes in wind direction, atmospheric stability, tidal inundation, and the relative contribution of channels, open water, or exposed sediment may still influence the measured flux [13,54,55]. Processing choices also contribute uncertainty. Low-turbulence filtering and the selected u* threshold affect nighttime NEE retention, while gap filling and flux partitioning influence the derived GPP and RE estimates [22,31,43,56]. The u* sensitivity analysis and monthly data-coverage assessment were therefore used to evaluate the robustness of the August pattern. These uncertainties should be considered together with the observed climatic and vegetation anomalies when interpreting the temporary positive-NEE episode.

Spatial predictions should be considered as a way to extend relationships observed at the tower to the broader landscape. However, additional field observations, independent remote-sensing products, and longer flux records are still needed to validate these landscape-scale estimates. Nevertheless, the concurrence of tower-based NEE, SPI-3, NDVI, and other environmental observations provides context for the August anomaly but does not identify its cause. The remaining question is whether it was an isolated event or part of a recurring response to climate variability.

## 5. Conclusions

The Del Carmen mangrove tower footprint was a net carbon sink during most of the March 2025–February 2026 study period, but this pattern was not constant. In August 2025, NEE shifted toward positive values during mild SPI-3 drought conditions within the *Habagat* season, indicating a temporary period of net CO_2_ release within the tower footprint. Since the record covers only one annual cycle, this event cannot be interpreted as evidence of a long-term trend, climate-change signal, or emerging new normal. It is better viewed as a short-term anomaly that demonstrates the sensitivity of mangrove CO_2_ exchange to changing environmental conditions. Nevertheless, the August 2025 episode shows that net CO_2_ uptake within the Del Carmen tower footprint can weaken temporarily under some environmental conditions, highlighting the need for longer-term observations before broader conclusions are drawn. The critical question is therefore no longer simply whether mangroves store carbon, but whether their sink strength will remain stable as climate extremes intensify. Longer-term measurements are needed to determine whether similar events recur and whether they have a meaningful effect on annual or multi-year carbon balance.

## Figures and Tables

**Table 1:** Monthly data coverage, gap-related uncertainty, and the number of days with negative NEE (net carbon uptake) and positive NEE (net carbon release) from March 2025 to February 2026.

Year	Month	Monthly Data Coverage					Days Carbon Uptake/Release	
		Expected Days	Valid Days	Total 30-Min Data	Total Data Gaps	% Data Gaps	Days NEE < 0 (Net CO_2_ Uptake)	Days NEE > 0 (Net CO_2_ Release)
2025	March	31	15	720	115	16	14	1
	April	30	30	1440	63	4	30	0
	May	31	31	1488	315	21	31	0
	June	30	30	1440	131	9	25	5
	July	31	31	1488	37	2	24	7
	August	31	31	1488	297	20	15	16
	September	30	30	1440	187	13	25	5
	October	31	31	1488	276	19	20	11
	November	30	30	1440	369	26	25	5
	December	31	31	1488	459	31	28	3
2026	January	31	31	1488	541	36	26	5
	February	28	18	864	329	38	17	1
**Overall**		**365**	**339**	**16,272**	**3119**	**19**	**280**	**59**

**Table 2:** Monthly summaries of carbon fluxes from March 2025 to February 2026, including mean daily fluxes with lower and upper 95% confidence limits and monthly cumulative sums. March includes 15 of 31 valid days (48.4% coverage), and February includes 18 of 28 valid days (64.3% coverage); therefore, monthly sums for these partial months represent available observations only and are not gap-filled estimates for the complete month. Confidence limits for mean daily fluxes were estimated using Monte Carlo bootstrapping with 1000 resamples and 95% percentile confidence limits.

Year	Month	Daily Average Fluxes (g C m^−2^ d^−1^)						Monthly Sum (g C m^−2^ Month^−1^)					
		GPP		RE		NEE		GPP		RE		NEE	
		Mean	*Upper Limit*	Mean	*Upper Limit*	Mean	*Upper Limit*	Mean	*Upper Limit*	Mean	*Upper Limit*	Mean	*Upper Limit*
			*Lower Limit*		*Lower Limit*		*Lower Limit*		*Lower Limit*		*Lower Limit*		*Lower Limit*
2025	March	5.14	*5.82*	2.19	*2.76*	−2.95	*−2.43*	77	*87*	33	*41*	−44	*−36*
			*4.36*		*1.62*		*−3.51*		*65*		*24*		*−53*
	April	6.03	*6.73*	3.19	*3.77*	−2.84	*−2.51*	181	*202*	96	*113*	−85	*−75*
			*5.36*		*2.55*		*−3.15*		*161*		*76*		*−94*
	May	5.25	*5.64*	2.14	*2.36*	−3.11	*−2.75*	163	*175*	66	*73*	−96	*−85*
			*4.85*		*1.89*		*−3.49*		*150*		*58*		*−108*
	June	5.47	*6.46*	3.31	*4.13*	−2.16	*−1.44*	164	*194*	99	*124*	−65	*−43*
			*4.57*		*2.54*		*−2.79*		*137*		*76*		*−84*
	July	5.17	*6.10*	3.81	*4.77*	−1.36	*−0.78*	160	*189*	118	*148*	−42	*−24*
			*5.20*		*2.89*		*−0.72*		*129*		*90*		*−61*
	August	5.11	*6.09*	5.71	*7.29*	0.60	*2.02*	158	*189*	177	*226*	19	*62*
			*6.09*		*4.36*		*−1.95*		*130*		*135*		*−22*
	September	6.98	*8.15*	4.64	*5.37*	−2.34	*−1.24*	209	*244*	139	*161*	−70	*−37*
			*5.85*		*3.88*		*−3.40*		*175*		*116*		*−102*
	October	5.10	*6.19*	3.90	*4.78*	−1.20	*−0.35*	158	*192*	121	*148*	−37	*−11*
			*4.06*		*3.09*		*−2.07*		*126*		*96*		*−64*
	November	5.37	*6.20*	3.78	*4.32*	−1.59	*−0.72*	161	*186*	113	*130*	−48	*−22*
			*4.58*		*3.23*		*−2.29*		*137*		*97*		*−69*
	December	7.82	*8.51*	5.40	*5.94*	−2.42	*−1.88*	242	*264*	167	*184*	−75	*−58*
			*7.10*		*4.83*		*−2.87*		*2220*		*150*		*−89*
2026	January	8.93	*9.88*	6.82	*7.57*	−2.11	*−1.46*	277	*306*	211	*235*	−66	*−45*
			*7.84*		*6.13*		*−2.70*		*243*		*190*		*−84*
	February	8.97	*10.24*	6.27	*7.25*	−2.70	*−2.11*	161	*184*	113	*131*	−49	*−38*
			*7.72*		*5.20*		*−3.27*		*139*		*94*		*−58*
**Overall**		**6.23**	***6.54***	**4.29**	***4.57***	**−1.94**	***−1.67***	**2113**	***2245***	**1455**	***1565***	**−658**	***−568***
			***5.92***		***4.01***		***−2.19***		***1982***		***1337***		***−739***

**Table 3:** Environmental associations with daily NEE, GPP, and RE for the full study period and August 2025. Thirteen initial predictors were first screened for multicollinearity using a VIF threshold of 5. PPFD and VPD were removed, leaving 11 candidate predictors. The remaining predictors were then evaluated separately for each response variable using bidirectional stepwise AIC selection. Predictors retained by AIC were included in the GAMs. Model performance was evaluated using adjusted R^2^ and temporally blocked cross-validation RMSE.

Period	Response	n	Predictor Retained After VIF Screening and AIC Selection	Adjusted R^2^	Blocked CV RMSE
March 2025 to February 2026	NEE	257	Rainfall, relative humidity, albedo, soil temperature, soil water content, high tide	0.38	2.53
	GPP	257	Soil temperature, albedo, rainfall, wind speed, soil water content, LAI	0.20	2.61
	RE	257	Relative humidity, net radiation, soil temperature, LAI, rainfall, albedo	0.19	2.44
August 2025 Only	NEE	31	Air temperature, wind speed, low tide	0.61	2.26
	GPP	31	Soil temperature, air temperature, relative humidity, soil water content, low tide	0.72	2.20
	RE	31	Wind speed, low tide, relative humidity, soil temperature, LAI	0.60	2.75

Note: PFD and VPD were removed during sequential VIF screening because of multicollinearity with the remaining environmental variables. Model relationships are interpreted as statistical associations rather than causal effects. GPP and RE are partitioned estimates derived from NEE and should therefore not be considered independent flux measurements.

## Data Availability

The analyses in this study are based on PhilFlux eddy covariance and environmental datasets, together with supporting gridded environmental products. The PhilFlux data are not currently available in a public repository but may be obtained from the corresponding author upon reasonable request, subject to applicable data-use and access conditions. The supporting gridded products are available from their respective data providers, as cited in the Section 2.
